# The Effect of Anti-Inflammatory Drugs on the Incidence of Colorectal Cancer

**DOI:** 10.3390/pharmaceutics18060643

**Published:** 2026-05-23

**Authors:** Marek Misiak, Aleksandra Maciejowska, Maciej Pałęga, Rafał Burek, Anita Gołda, Michalina Dworak, Beata Pawuła-Prgomet, Karol Forysiński, Tomasz Miłek

**Affiliations:** 1 Faculty of Medicine, Collegium Medicum WMCM, Cardinal Stefan Wyszynski University, 01-938 Warsaw, Poland; anita.goldaa@gmail.com (A.G.); tomasz_milek@wp.pl (T.M.); 2 Faculty of Medicine, Medical University of Gdańsk, 80-210 Gdańsk, Poland; 3 Collegium Medicum, Jan Kochanowski University in Kielce, 25-369 Kielce, Poland; 4 Faculty of Medicine, Medical University of Lublin, 20-093 Lublin, Poland; 5 Department of General Surgery and Oncology, Wolski Hospital Named After Dr Anna Gostyńska, Independent Public Health Care Facility, 01-211 Warsaw, Poland; beata.prgomet@gmail.com (B.P.-P.); forysinski@gmail.com (K.F.)

**Keywords:** colorectal cancer (CRC), nonsteroidal anti-inflammatory drugs (NSAIDs), aspirin (ASA)

## Abstract

**Background/Objectives:** Chronic inflammation is a key factor in the development and progression of colorectal cancer (CRC). When COX-2 levels and PGE_2_ production increase, nonsteroidal anti-inflammatory drugs (NSAIDs), including aspirin (ASA) and selective COX-2 inhibitors, such as celecoxib and rofecoxib, are commonly employed. This paper presents the effect of anti-inflammatory drugs, primarilyNSAIDs, on the incidence of CRC. **Methods:** A comprehensive literature search (119 articles) was conducted with databases such as PubMed. During our research, we used keywords such as colorectal cancer (CRC), nonsteroidal anti-inflammatory drugs (NSAIDs), ASA, COX, precision oncology, and personalized medicine. **Results:** The development of CRC is primarily associated with chronic inflammation and the actions of COX-2 and prostaglandin E2 (PGE_2_), which promote cancer cell proliferation and angiogenesis. Anti-inflammatory drugs act by inhibiting the secretion of COX-1 and COX-2 enzymes, which leads to reduced PGE_2_ production and may limit tumor growth. Aspirin has the best-documented and studied anti-cancer effect; long-term use is associated with a reduced risk of CRC development and mortality through its anti-inflammatory and antiplatelet effects, thereby limiting metastasis. Particularly beneficial effects are observed in patients with mutations in the PIK3CA gene. Factors influencing the effectiveness of CRC treatment include molecular differences and tumor location. **Conclusions:** The future of CRC treatment and prevention lies in personalized medicine, which accounts for each patient’s genetic profile. Decisions regarding NSAIDs use and CRC prevention should consider the potential benefits and risks of side effects.

## 1. Introduction

Colorectal cancer (CRC) represents one of the most common malignancies worldwide. In 2022, there were approximately 2 million new cases worldwide, a figure that has been increasing each year. CRC is the third most frequently diagnosed cancer, accounting for approximately 10% of all cancer cases and the second leading cause of cancer-related deaths [1,2,3]. Risk factors for colorectal cancer are divided into modifiable factors, including obesity, tobacco smoke, alcohol consumption or unhealthy diet, and non-modifiable factors, such as age over 50 years, male sex, inflammatory bowel disease (IBD) and genetic predisposition, most common Lynch syndrome (LS) and hereditary polyposis colorectal cancer (HPCC) [2,4,5]. One of the most important factors affecting prognosis is the location of the tumor, which is divided into right-sided colon cancer (RCC), covering the cecum to the proximal 2/3 of the transverse colon, and left-sided colon cancer (LCC), located from the distal 1/3 of the transverse colon to the rectum [6].

Chronic inflammation is a key factor in the development and progression of CRC, facilitating tumor cell proliferation, angiogenesis, and metastasis via the cyclooxygenase-2 (COX-2) and prostaglandin E2 (PGE_2_) pathways. When COX-2 levels and PGE_2_ production increase, nonsteroidal anti-inflammatory drugs (NSAIDs), such as aspirin, and selective COX-2 inhibitors, such as celecoxib and rofecoxib, are commonly employed; they inhibit COX-2 activity and reduce PGE_2_ production, thereby slowing tumor growth and inducing apoptosis in cancer cells [7].

A more detailed discussion of inflammation-driven carcinogenic mechanisms and associated signaling pathways is provided in Section 3.1.

Colorectal cancer is known to be one of many indications for aspirin, among such conditions as osteoarthritis, pain, cardiovascular risk reduction, angina pectoris prophylaxis, ankylosing spondylitis, rheumatoid arthritis, or systemic lupus erythematosus [8,9,10]. Aspirin is said not only to reduce the incidence of colorectal adenoma and colorectal cancer among high-risk persons (Lynch syndrome) [11], but also to improve disease-free survival after diagnosis, particularly among patients with tumors harboring somatic PIK3CA mutations [12].

In this area of research, many factors, such as genetic stratification or tumor location (colon cancer on the right side vs. the left side), should be taken into account [13]. Unfortunately, the reports to date contain many inaccuracies and contradictions, which provide a basis for summarizing the existing knowledge and indicating directions for further research.

The purpose of the study was to clarify the influence of different anti-inflammatory drugs, such as aspirin and COX-2 inhibitors. This review incorporates the most recent clinical data on NSAIDs in colorectal cancer and highlights existing inconsistencies in the literature, aiming to identify gaps and inform future research directions.

## 2. Materials and Methods

A comprehensive literature search was conducted with databases such as PubMed. This article ultimately included 119 studies (up to 2025). During our research, we used keywords such as colorectal cancer (CRC), nonsteroidal anti-inflammatory drugs (NSAIDs), ASA, COX, precision oncology, and personalized medicine. Only articles with valuable content relevant to the topic addressed in the article were included in the body of the work. All co-authors reviewed the available literature and drew upon it to create individual chapters.

## 3. Results

### 3.1. Carcinogenesis in Colorectal Cancer (Crc), Genetics, and Signaling Pathways

CRC is a highly heterogeneous disease, shaped by a complex interaction of genetic predispositions and environmental influences [14]. One of the key aspects of CRC pathogenesis is the emergence of benign precursor lesions, commonly referred to as polyps. The most significant risk for developing CRC is posed by adenomatous and serrated polyps, which are considered the most malignant [15,16].

CRC is characterized by dysregulation of multiple key signaling pathways, including Wnt/β-catenin, MAPK/ERK, PI3K/AKT/mTOR, TGF-β, Notch, Hedgehog, and JAK/STAT. Accumulation of mutations in genes such as APC, KRAS, BRAF, and PIK3CA leads to uncontrolled cell proliferation, impaired differentiation, inhibition of apoptosis, enhanced cell proliferation and invasion, and modulation of the tumor microenvironment [17]. Dysregulation of these pathways also underlies the development of polyp precursor lesions, promotes their progression to invasive cancer, and influences immune evasion, angiogenesis, and treatment resistance. This action renders them key mechanisms in the pathogenesis of both sporadic and hereditary CRC [18].

Polyps share a characteristic, defining feature of several hereditary syndromes predisposing to CRC, including familial adenomatous polyposis (FAP), MUTYH-associated polyposis (MAP), and Gardner syndrome. However, CRC can also develop without polyposis, as exemplified by hereditary non-polyposis colorectal cancer (HNPCC), commonly known as Lynch syndrome (LS) [16,19].

This suggests that both polyp-dependent and polyp-independent pathways contribute to the heterogeneous mechanisms causing colorectal carcinogenesis. Also, the transition from normal colorectal epithelial cells to adenomatous lesions and directly to invasive CRC is a multistep process that typically takes approximately a decade, enabling primary prevention and the implementation of screening tests, which in turn improve prognosis and clinical outcomes [18,20]. Most signaling pathway disorders in colorectal tumor initiation lead to overexpression of cyclooxygenase-2 (COX-2), which plays a crucial role in CRC development and promotes the production of its primary metabolite, prostaglandin E2 (PGE_2_). This phenomenon increases cell proliferation, angiogenesis, and resistance to apoptosis [21,22].

Carcinogenesis is a complex, multi-stage process in which genetic and epigenetic changes gradually transform healthy cells into cancer cells [23]. Inflammation is a fundamental mechanism by which the body defends itself against threats. However, when the inflammatory response becomes chronic, an avalanche begins—the body produces excessive cytokines and pro-inflammatory mediators. This drives both the innate immune response and tumor development. Prolonged action of pro-inflammatory mediators changes the tissue environment. A microenvironment arises in which DNA damage occurs more easily, and disturbances in cell cycle control become increasingly severe. Particularly concerning from an oncological point of view is the inhibition of apoptosis, that is, the natural death of cells. Prostaglandins and thromboxanes play a vital role. Their synthesis depends on the activity of cyclooxygenase-2 (COX-2). When COX-2 is excessively active—often observed in preneoplastic and neoplastic tissues—the entire process of tumor formation accelerates. Cancer cells divide more rapidly and survive longer. CRC is an excellent example of how chronic inflammation and tumorigenesis are interconnected. This type of cancer develops based on long-term inflammation and clearly shows that COX-2 overexpression goes hand in hand with disease progression [24].

Since the tumor microenvironment comprises cancer cells, stroma (the supportive tissue comprising connective and vascular/blood supply), and leukocyte(s), there are constant communications between these cells and factors produced within this environment as a consequence of their interaction, stimulating each other. Cancer cells are often located in proximity to one another; the stroma provides pathways for cancer cell migration and a supportive infrastructure for cancer expansion/proliferation/survival under therapeutic interventions. In conjunction with their parthenogenetic capabilities and supportive stroma, the presence of neutrophils, as discussed, acts to create an additional pathway of influence for furthering the progression of cancer(s) through their products, causing oxidative stress and consequently increased chances of further DNA mutation [24].

COX-1 acts constantly and maintains tissue balance. COX-2 appears when cytokines and growth factors come into play—then it becomes the primary source of PGE_2_ during inflammation and in tumors. In colorectal cancer, COX-2 is highly overexpressed. This drives the production of PGE_2_, which stimulates cell proliferation, promotes angiogenesis, and suppresses immune responses within the tumor microenvironment. PGE_2_ acts through EP receptors and activates signaling pathways, including cAMP/PKA, NF-κB, and PI3K/AKT, linking chronic inflammation to mechanisms that support the development and migration of cancer cells [25].

Overexpression of COX-2 and PGE_2_ goes hand in hand with a more aggressive phenotype of colorectal cancer and a higher risk of metastasis. This is precisely why this pathway is a therapeutic target and supports the use of COX-2 inhibitors for prevention and treatment [26].

In colorectal tumors, the PI3K/AKT/mTOR route is almost always constitutively activated, usually because PIK3CA is mutated or PTEN is lost. The stuck switch speeds cell division, blocks cell death, fuels tumor advancement, or blunts the impact of targeted drugs. The pathway is a major driver of colorectal cancer [27].

PI3K adds a phosphate group to lipids in the cell membrane. The altered lipids recruit and activate AKT, which in turn activates mTOR. AKT plus mTOR control how much protein the cell builds, when it divides, and how it uses nutrients. In colorectal tumors, AKT and mTOR are often switched on for too long—the cells migrate through tissue and ignore the signals that typically force them to die [28]. Mutations in PIK3CA give PI3K a stuck accelerator, while loss of PTEN removes the brake. The combined effect locks AKT and mTOR in the “on” position. The tumor cell receives a constant signal to divide and remain alive, even within the hostile environment of the large bowel tumor mass [29]. Inflamed tissue, typical of long-standing ulcerative colitis or the tumor bed itself, releases NF-κB into the nucleus. NF-κB drives TNF-α, IL-6, or related cytokines into the extracellular space. Those cytokines raise the amount of signaling proteins and cooperate with PI3K/AKT/mTOR. Blood vessel sprouts form, and tumor cells divide next to the suicide program that would clear damaged cell remains, remaining silent. NF-κB and PI3K/AKT/mTOR talk back and forth—inflammatory stimuli push NF-κB to transcribe more cytokines plus more pathway components. The feedback loop amplifies the growth impulse and drives the tumor from an early polyp to invasive colorectal cancer [30].

### 3.2. Pharmacology Nonsteroidal Anti-Inflammatory Drugs (NSAIDs)

Non steroidal anti-inflammatory drugs (NSAIDs) belong to a group of medications widely used in clinical practice worldwide. They have well-established analgesic, anti-inflammatory, and antipyretic effects. An increasing number of studies also indicate their potential protective effect against severe conditions, including cancers and cardiovascular diseases [31].

The primary mechanism of action of these drugs is the inhibition of cyclooxygenase enzymes (COX-1 and COX-2) and the subsequent reduction in prostaglandin synthesis, especially prostaglandin E2 (PGE_2_) [32]. In the colon, PGE_2_ promotes epithelial cell proliferation, inhibits apoptosis, enhances angiogenesis, and suppresses antitumor immune responses. Inhibition of COX-1 in platelets also plays an important role, leading to reduced platelet aggregation and decreased A_2_ synthesis [33]. Platelets participate in the metastatic process by protecting circulating tumor cells from the immune system and facilitating their adhesion to the endothelium. Their inhibition may therefore limit colorectal cancer cells’ ability to form metastases [34]. NSAIDs also influence the tumor microenvironment by modulating immune responses. It has been demonstrated that inhibition of the COX-2/PGE_2_ axis may reduce the expression of immunosuppressive molecules (e.g., PD-L1) and increase the infiltration of CD8^+^ T lymphocytes, thereby promoting the elimination of tumor cells [35].

The best-documented antitumor effect has been observed for acetylsalicylic acid, whose long-term use is associated with a reduced risk of colorectal cancer incidence and mortality, particularly in individuals with colorectal polyps or hereditary syndromes predisposing to intestinal malignancies [36]. Other NSAIDs (e.g., ibuprofen, naproxen, diclofenac) exhibit similar mechanisms of action; however, their chemopreventive effects are less well supported by clinical evidence and are limited by the risk of adverse effects [37].

#### 3.2.1. Acetylsalicylic Acid (ASA, Aspirin)

Acetylsalicylic acid (ASA, aspirin) is a classic nonsteroidal anti-inflammatory drug widely used for its analgesic, antipyretic, and anti-inflammatory properties [38]. In addition, during long-term use, ASA exhibits significant antithrombotic effects [39]. The mechanism of action of aspirin is based on non-selective inhibition of the cyclooxygenase enzymes COX-1 and COX-2, leading to decreased synthesis of prostaglandins (mainly PGE_2_) and thromboxane A2 (TxA_2_) [40]. The anti-inflammatory mechanism of ASA primarily results from inhibition of COX-2, thereby reducing PGE_2_ production and attenuating inflammatory processes in tissues [41]. In contrast, the antiplatelet mechanism involves irreversible acetylation of COX-1 in thrombocytes, thereby decreasing TxA_2_ synthesis and producing a long-lasting inhibition of platelet aggregation [40]. An increasing body of evidence indicates that aspirin’s antiplatelet activity plays a key role in cancer prevention, particularly in colorectal cancer (CRC) [42]. Long-term inhibition of platelet aggregation by ASA may limit the formation of microthrombi that promote tumor progression, thereby contributing to its chemopreventive effect [43]. In the British CAPP2 study conducted in 2011, the impact of daily administration of high-dose aspirin was evaluated in individuals with Lynch syndrome (HNPCC), who are at increased risk of colorectal cancer. Participants received 600 mg of aspirin daily for at least 2 years. After a mean follow-up period of nearly 5 years, a significant reduction in the risk of colorectal cancer was observed—by approximately 60% compared with the placebo group—as well as a 55% reduction in the incidence of other HNPCC-associated cancers [44]. Similar conclusions were drawn in 2024 based on a study conducted in a Danish population. The analysis used nationwide Danish registries that covered individuals aged 40–70 years at baseline (1 January 1997) and followed them through 2018. The use of low-dose aspirin (75–150 mg) was evaluated with respect to continuity, duration, and cumulative dose, as well as continuous use of high-dose aspirin (500 mg). Cox proportional hazards regression models were applied to estimate adjusted hazard ratios (HRs) and 95% confidence intervals (CIs) for overall cancer risk and selected cancer sites [45]. Regular use of low-dose aspirin did not significantly affect overall cancer risk; however, use for ≥5–10 years was associated with at least a 10% reduction in risk for several cancers, including colorectal, rectal, esophageal, gastric, liver, pancreatic, small intestinal, head and neck, brain, meningioma, melanoma, thyroid, non-Hodgkin lymphoma, and leukemia [45]. Meta-analyses of cohort studies have shown that regular aspirin use reduced the risk of CRC, gastric cancer, breast cancer, and prostate cancer [46]. In addition, meta-analyses of randomized controlled trials (RCTs) demonstrated that aspirin use was associated with a protective effect on CRC risk. By combining evidence from cohort study meta-analyses and RCTs, consistent evidence was obtained for a protective effect of aspirin use on colorectal cancer risk [46]. Subgroup analyses indicated that frequent aspirin use was associated with an increased risk of lung cancer. Unfortunately, dose–response analyses revealed that high-dose aspirin use may increase the risk of prostate cancer [46]. Caution should therefore be exercised when prescribing high-dose aspirin or when it is used frequently, given its association with an increased risk of lung and prostate cancer [46]. Further studies are needed to confirm these findings and determine the minimum effective dose for cancer prevention [46]. It has also been observed that aspirin reduces the expression of the DNA repair proteins MCM6 and RRM2 in human colorectal cells [47]. Recent studies indicate that aspirin affects nearly all hallmarks of cancer [48]. Within the tumor, this drug limits cancer cell activity and disrupts the tumor microenvironment that promotes tumor development [49]. In patients with hotspot PIK3CA mutations in exon 9 or 20, aspirin significantly reduced the risk of colorectal cancer recurrence compared with placebo, and similar benefits were also observed in individuals with other somatic alterations in PI3K pathway genes [12].

#### 3.2.2. Ibuprofen

Ibuprofen is an NSAID widely used to treat mild to moderate pain, inflammation, and fever [50]. Its mechanism of action is based on reversible inhibition of cyclooxygenase (COX) enzymes COX-1 and COX-2, thereby reducing the synthesis of prostaglandins (including PGE_2_ and PGI_2_) and thromboxane A2 [51]. The anti-inflammatory effect of ibuprofen results mainly from inhibition of COX-2, which reduces the production of prostaglandin E2 (PGE_2_) [52]. PGE_2_ plays a key role in carcinogenesis by promoting cancer cell proliferation, inhibiting apoptosis, enhancing angiogenesis, and suppressing immune responses [53]. Reduced PGE_2_ synthesis may theoretically limit tumor initiation and progression, particularly in cancers associated with chronic inflammation [54]. The antiplatelet effect of ibuprofen is related to inhibition of COX-1 in platelets, leading to a transient, reversible reduction in thromboxane A2 synthesis and inhibition of platelet aggregation [55]. Attenuation of platelet activity may theoretically limit metastatic spread; however, with ibuprofen, this effect is limited and short-lived [51].

#### 3.2.3. Ketoprofen

The primary mechanism of action of ketoprofen involves non-selective, reversible inhibition of cyclooxygenase COX-1 and COX-2, resulting in reduced synthesis of prostaglandins and thromboxane A2 from arachidonic acid [56]. The anti-inflammatory effect of ketoprofen is relevant in the context of carcinogenesis, as chronic inflammation of the colon promotes the development of colorectal cancer [57]. With respect to colorectal cancer, data on ketoprofen is much weaker than those available for acetylsalicylic acid [58]. Although molecular mechanisms suggest a potential protective effect resulting from inhibition of COX-2 and PGE_2_, there is a lack of clear clinical evidence confirming the effectiveness of ketoprofen in the chemoprevention of this cancer [59]. A 2023 study suggests that ketoprofen induces cytotoxic effects in colorectal cancer cells, including cancer stem cell populations, by modulating PUM1 protein activity; it also induces apoptotic cell death via activation of the caspase-dependent pathway. PUM1 (Pumilio RNA-binding protein 1) belongs to the PUF family of RNA-binding proteins [60]. In addition, suppressive effects on colorectal cancer stem cells were observed, as evidenced by a decrease in both the size and number of colonospheres formed. The compound induced apoptosis, leading to activation of caspases 3 and 7 in the HCT116 cell line, as demonstrated by a caspase-3/7 assay and AO/EB staining. At the same time, no significant cytotoxicity was observed toward non-cancerous cell lines, either in cell viability assays or hemolysis tests [60]. Furthermore, treated cells showed a significant reduction in PUM1 expression and in markers characteristic of colorectal cancer stem cells compared with control samples [60].

#### 3.2.4. Naproxen

Naproxen may inhibit the initiation and progression of colorectal cancer, particularly under conditions of chronic intestinal mucosal inflammation [61]. Inhibition of platelet activity by naproxen may therefore limit colorectal cancer cells’ ability to survive in circulation and colonize distant tissues. Naproxen has been shown to reduce the expression of programmed death-ligand 1 (PD-L1) and to promote increased infiltration of type I effector lymphocytes within colorectal tumors [62]. Importantly, both reduced PD-L1 levels and increased infiltration of CD8^+^ T lymphocytes correlated with inhibition of the COX-2/PGE_2_ axis, as demonstrated in in vitro studies and in syngeneic colorectal cancer xenograft models [35]. These findings indicate that naproxen may modulate immune responses within the tumor microenvironment, in part by attenuating immunosuppressive mechanisms associated with immune checkpoint signaling. For this reason, the potential role of naproxen as a chemopreventive agent is being considered for patients with colorectal polyps that exhibit positive PD-L1 expression [35]. Epidemiological and preclinical studies suggest that long-term use of naproxen may be associated with a reduced risk of developing colorectal cancer and with inhibition of premalignant lesion growth; however, these data are less consistent than those reported for acetylsalicylic acid [63].

#### 3.2.5. Diclofenac

Diclofenac is used in clinical practice primarily as an analgesic, anti-inflammatory, and antipyretic drug. Due to its pharmacological properties and its ability to modulate inflammatory and immune processes, it has been investigated for its potential impact on carcinogenesis, including colorectal cancer [64]. Diclofenac strongly inhibits COX-1 and COX-2; however, it does not exhibit significant cytotoxicity against colorectal cancer cells [64]. Conjugation of diclofenac or its structural motifs with other chemical groups, such as carboranes and oxindole structures, leads to the formation of prodrugs with markedly enhanced anticancer activity [65]. These modifications alter the compound’s biological properties, enabling anticancer effects not only through COX inhibition but also via COX-independent pathways [65]. The most active carborane derivatives strongly inhibited the proliferation of colorectal cancer cells and induced moderate apoptosis, accompanied by reduced levels of reactive oxygen and nitrogen species (ROS/RNS) [65]. These findings indicate that diclofenac, when combined with other chemical structures, may serve as a carrier molecule or pharmacophore whose structural redesign enables the development of compounds with selective and enhanced anticancer activity [65]. In a 2021 study, diclofenac was shown to reduce expression of the adhesion molecules CD44 and ICAM-1. Activation of COX-2 in the HT29 cell line using PMA resulted in a significant limitation of the increase in polyunsaturated fatty acid (PUFA) levels. Concurrent treatment with diclofenac and PMA led to a marked increase in apoptosis and enhanced caspase-3 activity in colorectal adenocarcinoma cells compared with untreated cells [66].

#### 3.2.6. Indomethacin

Indomethacin—another nonsteroidal anti-inflammatory drug—may reduce the incidence of colorectal cancer [67]. In the study, the anticancer properties of juglone in combination with indomethacin were evaluated in human colorectal adenocarcinoma HT29 cells [68]. Both morphological analysis and cell cycle assessment, along with AO/EB staining, confirmed the induction of apoptosis in treated cells [68]. The therapy led to decreased levels of the anti-apoptotic protein Bcl-2 and inflammatory mediators such as TNF-α, NF-κB, and COX-2, alongside increased expression of pro-apoptotic factors, including Bad, Bax, cytochrome c, and PUMA [68]. These effects were associated with modulation of the Wnt, Notch, and PPAR-γ signaling pathways. Although juglone alone exhibited weaker activity than indomethacin, their combination resulted in a stronger anticancer effect, indicating the potential utility of juglone as a component of combination therapy for colorectal cancer [68]. Another study demonstrated that indomethacin acts via activation of the GADD45α pathway and induction of apoptosis [69].

#### 3.2.7. Sulindak

Sulindac sulfone is a metabolite of the nonsteroidal anti-inflammatory drug sulindac that itself does not exhibit anti-inflammatory activity [70]. Clinical studies have shown that sulindac sulfone significantly reduces polyp counts in patients with familial adenomatous polyposis, suggesting its potential for cancer chemoprevention with minimal NSAID-related side effects. Sulindac sulfide inhibits the proliferation of colorectal cancer cells and reduces the expression of selected transcription factors, including Sp1, Sp3, and Sp4 [71]. In addition, several key regulators involved in cellular proliferation and survival are affected, including survivin, Bcl-2, the epidermal growth factor receptor (EGFR), cyclin D1, the p65 subunit of NF-κB, and vascular endothelial growth factor (VEGF). These molecules play essential roles in promoting cell growth, resistance to apoptosis, and angiogenesis [71].

*Preferential COX-2 Inhibitors*

#### 3.2.8. Meloxicam

Meloxicam is effective against colon cancer cells, reducing HT-29 cell viability and proliferation in an in vitro model [72]. It is used as an analgesic in the treatment of pain and inflammation associated with colon cancer. For this reason, a colon-targeted meloxicam delivery system has been developed, thereby increasing its effectiveness against colon cancer. Regular use of COX-2 inhibitors such as meloxicam is associated with a lower incidence and mortality from colorectal cancer (CRC) [73]. A significant reduction in viable HT-29 cells was observed at 24 and 48 h after exposure to meloxicam [72]. In the “scratch wound” test, inhibition of tumor cell migration was observed in HT-29 cells exposed to meloxicam, with effects that were concentration-dependent [72]. In an in vitro model, meloxicam induced morphological changes in HT-29 cells, suggestive of apoptosis or tumor cell damage [72]. Meloxicam, a member of the oxicam class, demonstrated a greater inhibitory effect than other NSAIDs in this study [72]. In a classic in vitro/in vivo model, meloxicam inhibited colony and tumor growth in the HCA-7 cell line. It reduced COX-2 expression, which is overexpressed in colorectal cancer and is associated with its carcinogenesis [74]. However, it did not affect the COX-2-negative HCT cell line, suggesting that its action may depend on the presence of COX-2 in tumor cells [74].

#### 3.2.9. Nimesulide

Nimesulide, a selective COX-2 inhibitor, reduced the incidence of dysplasia/neoplastic disease in a mouse model. In cell and animal models, it exhibits antiproliferative activity, induces apoptosis, and reduces the secretion of proangiogenic factors, such as VEGF, in colon cancer cell lines [75]. An in vitro study using the HT-29 cell line demonstrated that nimesulide at concentrations ranging from 10 to 1000 μmol/L inhibited HT-29 cell proliferation in a dose-dependent manner. Nimesulide increased the percentage of apoptotic cells. In vivo studies in a mouse model with HT-29 xenografts demonstrated 82.3% inhibition of tumor growth with HAL-nimesulide and 76.4% with HAH-nimesulide. Using nimesulide conjugate with hyaluronic acid improves its solubility and enhances the targeting of nimesulide delivery to neoplastic tumors [76]. The combination of nimesulide with 5-aminosalicylate resulted in more potent inhibition of HT-29 proliferation compared to nimesulide alone. A dose–response and time–response relationship was demonstrated. It should be noted that this study only involved an in vitro model [77]. Nimesulide exhibits antiproliferative effects on colon cancer cells. It has chronic effects in vitro, with mechanisms that include cell cycle blockade at G1 and induction of cyclin-dependent kinase inhibitors [78] (pp. 21, 27). Exposure of CRC cells to nimesulide induced an increase in the levels of cell cycle-inhibiting proteins. p21^Cip1 increased expression at the mRNA and protein levels, suggesting transcriptional modulation. p27^Kip1 increased protein levels in the absence of transcriptional upregulation, suggesting post-translational modulation [78].

#### 3.2.10. Etoricoxib

As a COX-2 inhibitor, etoricoxib, in models of chemical induction of colon cancer, may act against excessive COX-2 activity, which promotes the progression of precancerous and neoplastic lesions [79]. It significantly reduces the inflammatory potential of developing cancer by decreasing the expression of pro-inflammatory enzymes, inhibiting NF-κB activation, and normalizing the expression of other inflammation-dependent factors [80]. It demonstrates chemopreventive and antitumor effects in a DMH (dimethylhydrazine)-induced colon cancer model and in an early-stage CRC rat model by promoting tumor cell apoptosis, increasing the expression of GSK-3β, and downregulating the oncogenic PI3K/Akt pathway [80,81]. The effect of orally administered etoricoxib on rat models of DMH-induced colon cancer was studied. The expression of VEGF (vascular endothelial growth factor) was significantly increased in the DMH group and decreased after etoricoxib administration. The expression of MMP-2 and MMP-9 (metalloproteinases promoting matrix degradation and tumor progression) was increased dramatically in the DMH group and significantly reduced after etoricoxib treatment [80]. Anti-inflammatory drugs, including etoricoxib, modified the chemokine levels of MCP-1 and MIP-1β. MCP-1 was significantly reduced, and MIP-1β was increased considerably following their administration [80]. In an experimental model, in which etoricoxib was part of a combination of anti-inflammatory drugs, a reduction in the levels of angiogenesis markers (VEGF, MMP-2, MMP-9) was demonstrated, indicating inhibition of tumor-promoting vessel formation, promotion of tumor cell apoptosis, and modulation of the expression of chemokines associated with the tumor microenvironment, ultimately contributing to the chemoprevention of colorectal cancer in a DMH-induced tumor model [80,82].

*Selective COX-2 Inhibitors (Coxibs)*

#### 3.2.11. Celecoxib

In an in vitro study, celecoxib combined with ADT-OH demonstrated synergistic inhibition of HCT116 cell growth and reduced their migration capacity [83]. The combination of celecoxib with ADT-OH caused cells to arrest in the G0/G1 phase of the cell cycle [83]. Intracellular reactive oxygen species (ROS) levels also increased, leading to increased apoptosis [83].

A study of a nanovesicular form of celecoxib with altered surface charge was conducted in Wistar rats with colorectal cancer chemically induced with DMH (1,2-dimethylhydrazine). It was found that the bioavailability of celecoxib in the colon was improved in this formulation, approximately 2.13-fold compared to standard celecoxib suspension [84]. The nanovesicular formulation was also shown to have a more potent antitumor effect than conventional celecoxib [84]. In a clinical trial, patients with metastatic colorectal cancer demonstrated a significantly higher ORR (Objective Response Rate) after treatment with celecoxib compared to the control group. After three months of therapy, the celecoxib group showed considerably lower levels of VEGF, CXCL5, and sFASL. A significant increase in the pro-apoptotic marker sFAS was also observed, indicating facilitated apoptosis of tumor cells after administration of celecoxib in conjunction with standard anticancer therapy [85]. It was also found that one-year overall survival increased in the celecoxib group. The study also assessed the adverse events of celecoxib according to CTCAE V.6.0 and found that the combination was well tolerated in patients and did not cause severe toxicity [85].

#### 3.2.12. Rofecoxib

Like celecoxib, rofecoxib lowers prostaglandin levels by selectively inhibiting COX-2. Potentially protecting against colorectal neoplasia, rofecoxib exposure was associated with a reduced risk of developing colorectal polyps and neoplasms, suggesting a possible chemopreventive effect [86]. Randomized clinical trials and studies of rofecoxib as adjuvant therapy in CRC did not demonstrate significant improvement in survival or reduction in recurrence compared to placebo. It did not statistically improve disease response or disease control rates in patients with advanced colorectal cancer. Rofecoxib did not demonstrate a significant effect on 3-year survival; the benefit was primarily observed with celecoxib [87,88]. Rofecoxib use was associated with an increased risk of cardiovascular events in patients with colorectal adenomas or in the adjuvant setting in CRC [89,90]. Rofecoxib shows promise in the chemoprevention of sporadic colorectal cancer.

#### 3.2.13. Valdecoxib

The effect of valdecoxib on the biochemical properties of lipids in colon cancer cells was studied. It was hypothesized that the hydrophobic nature of valdecoxib might alter the physical parameters of lipids in cell membranes. Valdecoxib affects cellular lipids; after exposure to HT29 and SW620 cells, a reduction in lipid fluidity and changes in lipid ordering and dynamics were observed. This effect occurred regardless of COX-2 expression—it happened in both COX-2-positive and COX-2-negative cells. These lipid changes suggest that valdecoxib may exert therapeutic effects on colon cancer through a mechanism distinct from classical COX-2 inhibition [91].

#### 3.2.14. Parecoxib

Parecoxib is clinically used for parenteral administration; previous studies suggest that it may inhibit colon cancer cell metastasis through molecular mechanisms [92]. The combination of parecoxib (3 µM) with 5-fluorouracil (5-FU) significantly inhibited the migration and invasion of DLD-1 and SW480 cells, more so than either drug alone. Analysis demonstrated a synergistic effect in limiting cell migration [92].

Parecoxib treatment reduced the expression of β-catenin, a key factor in Wnt/β-catenin signaling that promotes EMT and tumor aggressiveness; it decreased vimentin levels, reduced Akt phosphorylation, increased the expression of GSK2β, which inhibits β-catenin, and increased the expression of E-cadherin [93]. Studies have shown that parecoxib inhibits EMT and migration of colon cancer cells by reducing Wnt/β-catenin signaling, limiting Akt phosphorylation, and modulating epithelial and mesenchymal markers [93]. In a clinical trial, 60 patients undergoing laparoscopic resection of rectal cancer were randomly assigned to two groups: a study group that received parecoxib sodium 40 mg intravenously at induction of anesthesia, immediately after surgery, and 12 h postoperatively, and a control group that received saline at the same time points [93]. Significantly lower serum levels of IL-6, TNF-α, and CXCL8 (indicators of inflammation) were observed after parecoxib administration compared to the control group. There was also lower expression in PBMCs (peripheral blood mononuclear cells): CXCL8, CXCR1, and CXCR2 chemokine-binding proteins and their receptors. This suggests that parecoxib may modulate the inflammatory microenvironment surrounding the tumor by reducing pro-inflammatory chemokines and receptors implicated in leukocyte migration [93].

In conclusion, parecoxib may improve the tumor microenvironment after rectal cancer surgery by reducing inflammation and CXCL8-CXCR1/2 receptor expression in peripheral blood cells [93,94].

*Other Drugs Affecting the Prostaglandin Pathway (Indirectly)*

#### 3.2.15. Glucocorticosteroids (GCS)

In vitro studies have shown that dexamethasone alters the rate of CRC cell migration depending on the level of GR receptor expression in HCT116 and HT29 cells. Protein-protein and ChIP-seq analyses revealed interactions between glucosteroid receptors and TET proteins in HEK293T cells, suggesting a receptor–epigenetic coupling in the mechanism of action of GCs. In an in vitro study, the drug Belinostat abolished the GCS-mediated invasion-promoting effect in CRC cells and reduced the expression of metastasis-associated genes [95]. An in vivo study in mice demonstrated that dexamethasone therapy significantly reduced colonic tumor growth compared with controls. In the same study, the number of tumors did not decrease completely, but the severity of histological changes decreased significantly. Dexamethasone decreased the expression of PCNA and cyclin D1 markers in colonic tissues. Dexamethasone reduced the infiltration of inflammatory mucosal cells and the activity of the MAPK/JNK pathway, especially with early intervention with dexamethasone [96]. In an in vivo study, GCS reduced the proliferation of HCT8/E11 cells and decreased the expression and activity [97].

#### 3.2.16. Paracetamol (Acetaminophen)

The effects of acetaminophen on proliferation, apoptosis, and apoptosis-related protein levels were studied in human colon cancer cell lines HT-29 and SW480. Acetaminophen caused only minor changes in proliferation in proliferating HT-29 and SW480 cells. It increased apoptosis in both cell lines (HT-29 and SW480), both alone and in combination with metamizole. In SW480 cells, it is associated with reduced expression of caspase 3 and caspase 8 (proteins associated with apoptosis) [98].

The effect of AM404, a metabolite of acetaminophen, on colon cancer was also studied. AM404 is an anandamide uptake inhibitor with antibacterial activity [99]. AM404 has strong potential to inhibit CRC stem cell characteristics, including stemness/dedifferentiation, migration, and treatment resistance [99]. AM404 inhibited FBXL5 expression, which may contribute to colon cancer invasion and chemotherapeutic resistance [99].

*Other Drugs*

#### 3.2.17. Sulfasalazine

Sulfasalazine is a prodrug that is cleaved in the intestinal lumen by bacterial enzymes to yield the active moiety 5-aminosalicylic acid (5-ASA) and sulfapyridine. 5-ASA acts as a local anti-inflammatory by altering the cyclooxygenase and lipoxygenase pathways, inhibiting prostaglandin and leukotriene synthesis at the level of intestinal mucosa, thus modulating inflammation in inflammatory bowel diseases [100].

Studies in recent years show that 5-ASA can also produce anti-inflammatory effects beyond the intestine, such as inhibiting COX-2-dependent pro-inflammatory responses in several models of osteoarthritis, implying a widespread activity of sulfasalazine metabolites on COX pathways [100]. Acetylsalicylic acid (aspirin), a metabolic product of salicylates, irreversibly acetylates the COX-1 isoenzyme and modulates the activity of COX-2, consequently preventing pro-inflammatory prostanoid biosynthesis and PGE_2_ production. This is an important mechanism underlying its anti-inflammatory and antiplatelet actions [101].

#### 3.2.18. Mesalazine (5-Aminosalicylic Acid, 5-ASA)

Mesalazine is the first-line treatment for both inducing and maintaining remission in mild to moderate ulcerative colitis. In accordance with the procedure of Eder et al. (2023) [102], its pharmacotherapeutic activity mostly depends on the local suppression of inflammatory reactions within the colonic mucosa by modifying COX-dependent pathways and decreasing prostaglandin production. The safety profile of the drug is good, and long-term use, including maintenance therapy, is possible. The guidelines focus on the role of route of administration, noting that combination therapy (oral and rectal) enhances treatment efficacy, particularly in distal colitis. Mesalazine is still an essential basis for treatment of ulcerative colitis in symptom reduction and maintenance therapy of remission [102].

### 3.3. Pharmacology Summary and Estimation of CRC Risk in ASA Users vs. Control Groups

An enzyme, cyclooxygenase-2 (COX-2), is known to be overexpressed in colorectal cancer. This suggests that COX-2 inhibition may have antineoplastic effects. Using aspirin or other COX inhibitors proved beneficial.

A. Martling et al. (2025) [12] conducted a double-blind, randomized, placebo-controlled trial involving patients receiving 160 mg of aspirin or a matched placebo once per day with stage I, II, or III rectal cancer or stage II or III colon cancer with somatic alterations in PI3K pathway genes. Authors focused on patients with PIK3CA hotspot mutations in exon 9 or 20 (group A alterations) and other moderate- or high-impact somatic variants in PIK3CA, PIK3R1, or PTEN (group B alterations). After 3 years, the conclusion was reached that aspirin was associated with a lower incidence of colorectal cancer recurrence among patients with PIK3CA hotspot mutations in exons 9 or 20 and appeared to have a similar benefit among those with other somatic alterations in PI3K pathway genes [12].

It is also suggested that aspirin may limit metastasis by inhibiting platelet activation via COX-1 and COX-2, through an independent PI3K signaling mechanism that may enhance immune clearance of circulating tumor cells [12,103].

Long-term use of low-dose aspirin lowers the chance of developing colorectal cancer and of dying from it. The drug blocks the enzymes COX-1 and COX-2, thereby reducing the production of prostaglandins that drive inflammation. Agents that block only COX-2 were designed to spare COX-1, therefore causing fewer side effects. Population studies show that aspirin and COXibs slow the growth of precancerous lesions, but the data for COXibs are less convincing. In addition, coxibs raise the risk of heart attack but also stroke—they are not suitable for extended use. For its part, it carries a higher risk of bleeding in the stomach or gastrointestinal tract. Any decision to use drugs to prevent colorectal cancer must weigh the possible benefits against those concrete harms [104].

Aspirin and the selective COX-2 inhibitors shut down the cyclooxygenase enzymes—the drop in PGE_2_ slows the division of tumor cells, nudges them toward death plus chokes off new blood vessel growth inside the tumor. Aspirin and COXibs also slip past the COX blockade but also tamper with intracellular signals like NF-κB, altering cell growth, cell death, and immune reactions. The combined hit on the COX-2/PGE_2_ circuit plus those separate pathways gives a layered attack on the tumor as well as underpins the use of aspirin and COXibs to prevent or treat colorectal cancer [105].

Among non-selective NSAIDs, aspirin is shown to be the strongest and most consistent evidence for CRC chemoprevention, whereas other agents demonstrate weaker or less consistent clinical effects, often limited by safety profiles or insufficient clinical validation. Despite coxibs mechanistic specificity, selective COX-2 inhibitors are limited in long-term chemopreventive use because of the increased cardiovascular risk, which reduces their clinical applicability compared to aspirin.

A comparative evaluation of NSAIDs indicates that aspirin remains the most evidence-supported agent for CRC prevention, while selective COX-2 inhibitors and other NSAIDs show more limited or context-dependent efficacy, primarily due to safety concerns and weaker clinical validation.

### 3.4. CRC Tumor Properties Depend on Its Location (Right/Left Side)

Differences in embryonic origin, vascular and nervous supplies, microbiota burden, and primary physiological functions of the left and right colons, as well as tumor location, are said to affect tumor pathology, progression, and prognosis [106]. Existing reports demonstrate different outcomes for patients with right-sided compared to left-sided colorectal cancer (CRC). According to Kavitha Mukund et al. (2020) [106], right-sided colon cancers arise in the cecum, ascending colon, hepatic flexure, and/or transverse colon. Left-sided colon cancers arise in the splenic flexure, descending, and/or sigmoid colon [106]. Additionally, differences in treatment response based on disease laterality have been observed. Right-sided colorectal cancer has a worse prognosis compared to left-sided CRC, which is driven by the complex biological diversity of these malignancies [107]. Genetic differences between right- and left-sided CRC have been described; the genetic and molecular drivers underlying differences in prognosis and treatment response remain poorly studied. Differences in gene expression between normal mucosa and adenocarcinomas of the caecum and sigmoid or rectosigmoid should be considered in future studies and in patient treatment [108]. Additionally, different prevalence patterns across age groups, in high- and low-incidence nations, and in men and women have been documented. RCCs are more common in females, whereas LCCs are more common in males [109]. According to Michael S. Lee et al. (2017) [110], right-sided colon cancers are more likely to exhibit genome-wide hypermethylation via the CpG island methylator phenotype (CIMP), a hypermutated state via microsatellite instability, and BRAF mutation. Biologic subtypes are differentially distributed between right- and left-sided CRCs, with greater proportions of the “microsatellite unstable/immune” CMS1 and the “metabolic” CMS3 subtypes found in right-sided colon cancers [110]. In 2018, Christopher E. Jensen et al. [111] reported that among 288 cases, patients with left-sided primaries had longer overall survival from pathologic diagnosis. At the same time, BRAF and CTNNB1 mutations were more prevalent in right-sided CRC. BRAF was mutated at 15.5% of right-sided CRC compared to 4.8%. CTNNB1 was mutated in 3.9% of right-sided CRC compared to no instances of CTNNB1 mutations in left-sided disease [111]. Mohamed E. Salem et al. (2020) [112] link the rising incidence of CRC, particularly left-sided tumors, in adolescents and young adults to epigenetic events. According to him, right-sided tumors showed higher mutation rates than LT in several genes, including BRAF (10.3% vs. 2.8%), KRAS (64.1% vs. 45.5%), PIK3CA (27% vs. 11.2%), and RNF43 (24.2% vs. 2.9%). Other mutations are in distinct genes involved in histone modification, chromatin remodeling, and DNA repair. Also, cancer-predisposing syndromes were characteristic of right-side tumors, most frequently KMT2D (27.8% vs. 3.4%), ARID1A (53.3% vs. 21.4%), MSH6 (11.1% vs. 2.3%), MLH1 (10.5% vs. 2.3%), MSH2 (10.5% vs. 1.2%), POLE (5.9% vs. 0.6%), PTEN (10.8% vs. 2.3%), and BRCA1 (5.4% vs. 0.6%). MSI was seen in 20.8% of right-side tumors versus 4.8% of left-side tumors. RT is associated with a higher frequency of TMB-high, regardless of MSI status [112]. Yu-Lun Hsu et al. (2019) reported that right-sided colon cancer had more gene mutations in BRAF, KRAS, SMAD4, TGF-β, PIK3CA, PTEN, and AKT1, and high microsatellite instability [113]. Kavitha Mukund et al. (2020) [106] highlight a nexus between calcium homeostasis (sensing, mobilization, and absorption) and immune/GPCR signaling within left-sided tumors, which may contribute to reduced proliferative and metastatic potential. Also, two genes, SLC6A4 and HOXB13, have been reported to exhibit opposing regulatory trends between right and left tumors. Post-transcriptional regulation mediated by both RNA-binding proteins (e.g., NKRF (in left) and MSI2 (in right)) and miRNAs (e.g., miR-29a (in left); miR-155, miR181-d, miR-576, and miR23a (in right)) appears to exhibit side-specificity in control of their target transcripts and is pronounced in right colon tumors. Researchers emphasized increased hypomethylation in open seas within left tumors and increased hypermethylation of CpG islands within right tumors [106]. Ohamedd E Salem et al. (2017) [114] found that right-sided colon cancers have higher rates of microsatellite instability, more frequent aberrant activation of the EGFR pathway, including higher rates of BRAF and PIK3CA mutations, and a higher mutational burden than left-sided colon and rectal cancers. At the same time, rectal cancers were said to have higher rates of TOPO1 expression and Her2/neu amplification compared to both left- and right-sided colon cancers [114]. Yayoi Takahashi et al. (2016) [115] performed a comprehensive molecular analysis using crypt isolation with samples from 92 sporadic CRCs. Microsatellite instability (MSI; high and low/negative) and DNA methylation status (low methylation epigenome; intermediate methylation epigenome [IME] or high methylation epigenome [HME]) were determined using polymerase chain reaction (PCR) microsatellite analysis and PCR-bisulfite pyrosequencing, respectively. Ninety-two CRCs were classified into 71 MSS and 21 MSI phenotypes. Mutations in KRAS were associated with RC with the MSS phenotype, whereas mutations in TP53 were more frequently found in LC with the MSS phenotype. There were significant differences in KRAS and TP53 mutation frequencies in the IME between LC and RC with the MSS phenotype. Although CNA gains were associated with LC with the MSS phenotype, CNA losses were not major alterations associated with the MSS phenotype. These findings suggested that the molecular pathogenesis of the MSS phenotype in LC was different from that in RC [115].

Tumor location (colon cancer on the right side vs. the left side) may also influence responsiveness to aspirin, probably through differing microenvironments and mutation profiles [13,116,117].

## 4. Conclusions

Chronic inflammation is widely recognized as a central driver of colorectal carcinogenesis, influencing tumor initiation, progression, immune evasion, and metastatic potential. This review synthesizes evidence on the role of anti-inflammatory drugs—particularly aspirin, non-selective NSAIDs, and selective COX-2 inhibitors—in reducing the incidence and recurrence of colorectal cancer (CRC), with emphasis on molecular mechanisms, tumor heterogeneity, and drug-specific limitations. Among all agents reviewed, aspirin demonstrates the most robust and clinically validated protective effect against CRC. Long-term aspirin use has been associated with reduced CRC incidence, lower recurrence rates, and improved disease-free survival, especially in genetically defined subgroups. Notably, patients with somatic alterations in the PI3K pathway—particularly PIK3CA hotspot mutations—derive a pronounced benefit from aspirin therapy, as demonstrated in randomized controlled trials. These findings underscore the importance of molecular stratification in identifying patients most likely to benefit from aspirin-based chemoprevention or adjuvant treatment. Beyond COX-2 inhibition, aspirin exerts antitumor effects through platelet inhibition via irreversible COX-1 acetylation. Platelets play a critical role in protecting circulating tumor cells, facilitating immune escape, and promoting metastatic dissemination. Thus, aspirin’s antiplatelet activity may be a key mechanism that limits CRC progression and metastasis, independent of its direct effects on tumor cells. This dual mechanism—anti-inflammatory and antithrombotic—likely explains aspirin’s superiority over other NSAIDs in CRC prevention. Non-aspirin NSAIDs such as ibuprofen, naproxen, ketoprofen, diclofenac, indomethacin, and sulindac share similar molecular targets but show less consistent clinical evidence for CRC prevention. While in vitro and animal studies suggest antiproliferative, pro-apoptotic, and anti-metastatic properties, their long-term use is limited by gastrointestinal, renal, and cardiovascular toxicity. Selective COX-2 inhibitors (coxibs) offer a more targeted approach to inflammation-driven carcinogenesis. The safety concerns highlight the delicate balance between anticancer efficacy and systemic toxicity. Emerging evidence suggests that drug efficacy may also depend on tumor sidedness, with biological differences influencing responsiveness to anti-inflammatory drugs, including aspirin. However, definitive conclusions require further prospective studies. The anti-inflammatory drugs described above are used for a variety of indications and may also reduce the incidence of colon cancer. Investigating the molecular profile of CRC patients is key to advancing personalized medicine and improving clinical outcomes.

## 5. Discussion

CRC remains a major global health burden highlighting the need for developing novel therapeutic strategies, particularly those targeting inflammation-driven carcinogenesis and also specific molecular pathways within the framework of personalized medicine. Some experimental studies have explored the anti-inflammatory potential of bioactive compounds derived from natural sources. Compounds isolated from *Moringa oleifera* seeds were shown to modulate inflammatory responses [118]. Such findings show valuable insights into potential future therapeutic approaches targeting the tumor microenvironment and inflammatory processes.

There is also emerging evidence suggesting that pharmacological agents like glucagon-like peptide-1 (GLP-1) may also influence key oncogenic pathways relevant to CRC. Liraglutide has been reported to modulate the PI3K/AKT/mTOR signaling pathway and affect cellular processes including proliferation, cell cycle, migration, invasion, and apoptosis [119] which seems to be a promising direction in CRC therapy.

Variability in pharmacokinetic profiles among NSAIDs can affect tissue distribution, local colonic exposure, and duration of cyclooxygenase inhibition, which may modulate their potential preventive effects in CRC. That may partly explain the observed heterogeneity in chemopreventive outcomes across individual NSAIDs.

Many observational studies lack precise control of confounding factors, while experimental findings often rely on in vitro or animal models with limited translational applicability. These limitations underscore the need for large, well-designed randomized trials that incorporate genetic, epigenetic, and tumor location variables. Future research should focus on personalized chemoprevention strategies, optimal dosing regimens that minimize toxicity, and integration of molecular profiling to identify patients most likely to benefit, using larger cohorts to confirm previously observed trends. Therefore, decisions regarding NSAID use and CRC prevention require medical consultation and appropriate patient screening, even when we use them for preventive purposes.

In the future, it will be crucial to develop precise, evidence-based clinical recommendations that will enable clinicians to make the most appropriate treatment decisions. The goal is to achieve anti-cancer effects while reducing the risk of side effects. It will improve the safety and efficacy of the treatment, potentially extending the survival of CRC patients.

## 6. Take-Home Message

- The development of this cancer is primarily associated with chronic inflammation and the actions of COX-2 and prostaglandin E2 (PGE_2_). These enzymes promote cancer cell proliferation, angiogenesis, and inhibit apoptosis. Anti-inflammatory drugs act by inhibiting the secretion of COX-1 and COX-2 enzymes, which leads to reduced PGE_2_ production and may limit tumor growth.
- Aspirin has the best-documented and studied anti-cancer effect; long-term use is associated with a reduced risk of CRC development and mortality through its anti-inflammatory and antiplatelet effects, thereby limiting metastasis. Particularly beneficial effects are observed in patients with mutations in the PIK3CA gene. Other NSAIDs have similar effects, but their clinical effectiveness is not as well studied.
- Selective COX-2 inhibitors also have potential for CRC prevention, but their side effects limit their use.
- Factors influencing the effectiveness of CRC treatment include molecular differences and tumor location.
- The future of CRC treatment and prevention lies in personalized medicine, which considers each patient’s genetic profile. Integration of molecular and clinical factors is essential for the development of personalized approaches to NSAID-based chemoprevention in colorectal cancer. Despite promising results, many studies have limitations and require further confirmation in clinical trials. Therefore, decisions regarding NSAID use and CRC prevention should consider their potential benefits and risks.

## Data Availability

No new data were created or analyzed in this study.

## Abbreviations

The following abbreviations are used in this manuscript:

CRC	Colorectal cancer
NSAIDs	Nonsteroidal anti-inflammatory drugs
PGE_2_	Prostaglandin E2
RCC	Right-sided colon cancer
LCC	Left-sided colon cancer
COX-2	cyclooxygenase-2
ASA	Aspirin, Acetylsalicylic acid
LS	Lynch syndrome
